# A preliminary validation of PMQ—A four-factor questionnaire measuring parental mentalizing

**DOI:** 10.3389/fpsyg.2024.1250092

**Published:** 2024-06-12

**Authors:** Timo Teräsahjo, Tiina Turunen, Oskari Lahtinen, Christina Salmivalli

**Affiliations:** Faculty of Psychology, University of Turku, Turku, Finland

**Keywords:** parental mentalizing, parental reflective functioning, assessment of parental mentalizing, scale development, confirmatory factor analysis

## Abstract

The present study describes the construction and preliminary validation of a new parental mentalizing scale, PMQ. Based on theory, we hypothesized that one higher-order parental mentalizing factor would comprise four dimensions of parental mentalizing: (1) Parental self-mentalizing (SELF), (2) Parental child-mentalizing (CHILD), (3) Effort (E), and (4) Curiosity (C). After modifying the content of one factor (Effort > Lack of Effort, LE), four-factor structure with one higher-order factor was confirmed in data collected from parents of children aged two to 6 years through social media and email lists (N = 321, 10% male). All factors loaded significantly on the higher-order factor, with acceptable internal consistencies. Next, PMQ factors were compared with the factors of a previously validated questionnaire, parental reflective functioning questionnaire (PRFQ). The PMQ and PRFQ factors were consistently and significantly correlated, indicating the validity of the PMQ as a measure of parental mentalization ability. The continuation of PMQ validation is discussed.

## Introduction

Mentalization, or mentalizing, is the ability to understand the mental state, both of oneself or others, underlying overt behavior (Bateman and Fonagy, 2016). The concept of parental mentalization refers to mentalizing in the context of parenting. It captures the parental capacity to represent their child as a psychological agent, and the parent’s proclivity to understand and interpret the child’s behavior in terms of mental states (Medrea and Benga, 2021). The parent’s capacity to mentalize has a positive effect on the sensitivity of caregiving and on the child’s attachment security (Zeegers et al., 2017). It affects the parent’s own (Schultheis et al., 2019) as well as the child’s emotion regulation (Senehi et al., 2018) positively and is connected to the development of the child’s capacity to reflect upon mental states (Meins et al., 2002). The importance of parental mentalizing has been widely recognized in the field, leading to numerous parental and family interventions based on mentalization theory (Midgley et al., 2021). However, measuring parental mentalizing is challenging since existing interviews are time- and labor-intensive and existing self-report questionnaires tap only to some aspects of mentalization and thus lack the ability to assess change in parental mentalization in the context of mentalization-based interventions, for example. Therefore, the aim of this study is to develop and validate a new parental mentalizing scale that would assess parental mentalizing in a comprehensive manner and be sensitive enough to capture change in mentalization ability even among parents with relatively good mentalization skills to begin with.

Mentalization develops in the context of the attachment relationship where a child observes, mirrors, and internalizes his or her attachment figure’s ability to represent and reflect mental states (Fonagy and Bateman, 2019). This process is closely connected to the development of emotion regulation (Luyten et al., 2020). In the dyadic parent–child regulation system, the child internalizes not only the parent’s ability to reflect mental states but also the parent’s capacity to hold the child’s emotions in an accepting and safe way. In order for this to happen, the parent must be able to see their child as an intentional subject with an inner world of their own (Medrea and Benga, 2021) and be able to respond to the child’s initiatives sensitively and markedly (Milesi et al., 2023).

From the child’s perspective, the parental mentalizing offers experiences of “being recognized,” which are particularly crucial for their favorable development. The parent’s genuine interest in the child’s mental states opens the channel to what is called “epistemic trust:” the capacity to see others as trustworthy sources of knowledge that is generalizable and relevant to the self (Luyten et al., 2020; Milesi et al., 2023). Thus, the child learns not only to perceive their caregiver as a reliable source of information but also to benefit from positive influences in their environment more generally (Luyten et al., 2020). This characteristic of secure attachment relationship is often lacking in adverse childhood experiences and insecure attachment, which have strong and positive associations with epistemic mistrust and epistemic credulity (Li et al., 2023).

Parental mentalizing may be described as the capacity to create a certain kind of state of mind, referred to as *mentalizing awareness*, when interacting with a child. In daily life, it appears as a parent’s: basic ability to differentiate between internal and external reality; capacity to make observations of momentary changes in the child’s mental state; and when needed, to hold and contain the child’s experiences (Slade, 2005). Through this process, the child learns how the mind works, and they also learn that the mind of the other can serve not only as a pathway to closeness but also as a source of valuable information. The depth with which the social environment can ultimately be processed is learned through these interactions (Fonagy et al., 2002). This process is contrary to a psychopathological stance, wherein a person cannot enter fully into one’s own nor others’ subjective experience without reliance upon primitive defenses and distortions (Slade, 2005).

Mindfulness is a necessary prerequisite for mentalizing awareness (Allen, 2013; Török and Kéri, 2022). The acceptance of feelings and emotions, i.e., the containing function of mindfulness, makes mentalization-based emotion regulation possible (Török and Kéri, 2022). Similarly, heightened attention to and vivid moment-to-moment observation of one’s experiences are precursors of sensitivity in understanding all mental states in oneself and in others. Mindfulness, like mentalizing awareness, is a state of mind rooted in curiosity. Without genuine curiosity toward the child, the parent may be unable to enter into the internal subjective world of the child (Luyten et al., 2017). Effective mentalizing requires also sensitivity to understand misunderstandings, meaning the individual is attentive and responsive to breakdowns in understanding and connection, can actively process information and act to achieve understanding when needed. A parent who mentalizes effectively can switch flexibly to the kind of mentalizing that involves attention, awareness, intention, and effort (Luyten et al., 2017). The parent is then able to, for example, imagine what kinds of thoughts and feelings may be behind a child’s otherwise incomprehensible aggressive behavior. Effective parental mentalizing is not, however, only about the child because the parent’s ability to reflect on their own inner life affects how they understand their children and interact with them (Suchman et al., 2010).

Effective mentalizing can be conceptualized as a balance of four hypothetical underlying neural circuits: self–other, emotion–cognition, automatic–controlled, and internal–external (Luyten et al., 2020). Achieving equilibrium between the self–other dimension means the individual is capable of mentalizing their own state, including the individual’s own physical experience, and that of the *other* with flexibility and balance (Fonagy and Bateman, 2019). In the very same way, an individual’s ability to balance between emotion and cognition, without either side dominating, allows them to “feel clearly” (Choi-Kain and Gunderson, 2008). In everyday life, an effectively mentalizing individual does not need to reflect on everything; they can operate with the help of automatic models (i.e., automatic mentalizing) until faced with a problematic situation, requiring them to transition flexibly to a more conscious, verbal, and effortful analysis of mental states (i.e., controlled mentalizing). In the internal–external dimension, one must balance between understanding one’s own mind and that of others based on external features and through a direct focus on the inner experience. An imbalance in the internal–external dimension can appear, for instance, as excessive reliance on facial expressions or body positions when interpreting another’s state of mind. When mentalization is off-balance, for example, due to stress, a well-mentalizing individual can recover from a break in mentalizing relatively quickly, and the threshold for such a malfunction of mentalization ability may be high. Parental mentalizing capacity is distinct from general mentalizing capacity because mentalization is a context-dependent ability. However, these abilities are connected (Luyten et al., 2017).

Within the family, the parent may mentalize another child better than her sibling with a different temperament (Jovanc Ević et al., 2021), and there may be differences in parental mentalizing abilities between parents (Luyten et al., 2017; Madsen et al., 2023). Mentalizing profiles can also vary between cultures given that linguistic factors, value preferences, and parenting characteristics may affect how mentalization manifests itself in a certain cultural environment (Aival-Naveh et al., 2019). Within a culture, in turn, the social environment surrounding the parent–child dyad can have a significant effect on mentalization. For instance, social inequalities and experiences of powerlessness and inequality can affect mentalizing in the parent–child relationship (Campbell and Allison, 2022).

There are several definitions for parental mentalization in the research literature. *Parental reflective functioning* (Luyten et al., 2017) is defined as the capacity of the parent to envision their child as being motivated by internal mental states, to reflect on the parent’s own internal mental experiences, and to understand how these mental states are influenced by interactions with the child. *Maternal mind-mindedness* (Meins et al., 2002) signifies the parent’s ability to accurately reflect the child’s mental states during an interaction. It represents the parent’s capacity to be sensitive to what goes through the child’s mind (Medrea and Benga, 2021). *Parental embodied mentalizing* (Shai and Belsky, 2017) operationalizes parental mentalization in terms of non-verbal, bodily based, and interactive behavior where the caregiver manifests their understanding of the infant’s mental state via bodily gestures. *Maternal insightfulness* (Koren-Karie et al., 2002) refers to the parents’ capacity to consider the motives underlying their child’s behavior in a complete, positive, and child-focused manner. Also, *mindful parenting* (Ahemaitijiang et al., 2021) targets the parental mentalizing process as it extends mindfulness from the intrapersonal relationship to the interpersonal relationship. It refers to open, non-judgmental, and present awareness in one’s interaction with the child, including “an attempt to understand children’s thoughts and think from the perspective of children,” and “recognition and awareness of children’s inner selves,” (Ahemaitijiang et al., 2021).

Considering that the ability to mentalize is context-dependent and not a unitary but a multidimensional capacity (Fonagy and Bateman, 2019), it is quite clear that these different operationalizations may not encompass parental mentalization exhaustively. Different concepts measure diverse aspects of parental mentalizing ability and capture different processes related to the parent’s mental representation and behavioral competence (Medrea and Benga, 2021; Stuhrmann et al., 2022). For example, parental reflective function and parental insightfulness differentially emphasize how the parent reflects upon their child’s mind, while mind-mindedness is about parent’s meaning making and behavior during real-life interaction with the child (Medrea and Benga, 2021). Parental reflective functioning and mindful parenting differ from other operationalizations in that, in addition to understanding the child’s mind, they also take into account the parent’s reflection of their own mental states.

The research methodology of parental mentalization, as well as mentalization in general, has been mainly based in either observations or interviews, encoded with special coding systems. For example, parental reflective functioning is usually measured using a 45-item clinical semi-structured interview (Parental Development Interview, PDI), which is transcribed and then scored for parental reflective functioning (Medrea and Benga, 2021), while measurement of maternal mind-mindedness can be based on observation of free interaction between the caregiver and the child and coding of parent’s mind-related comments (Medrea and Benga, 2021). Examining mentalization with questionnaires has been discouraged by some as self-assessment may narrow the possibilities of measuring the parent’s mentalizing ability. For instance, measuring the parent’s actual capacity to hold, regulate, and fully experience emotion, a central component of effective mentalizing (Slade, 2005), is likely no more accessible with self-assessment questionnaires than IQ. In addition, successful adaptation of such a measure for various cultural environments is necessary and challenging (Aival-Naveh et al., 2019). Despite all this, measuring the complex concept of parental mentalizing, even with limited structural instruments, is useful. For example, the Parental Reflective Functioning Questionnaire, PRFQ, has already been demonstrated to be an efficient way to measure a parent’s capacity to recognize their child’s mental states and to understand the relationship between underlying mental states and behavior (Carlone et al., 2023).

While *The Handbook of Mentalizing in Health Practice* (Luyten et al., 2019) lists 19 questionnaires that can be used as proxy measures of mentalizing, most were originally developed for other purposes, and there is a lack of self-assessment tools for evaluating parental mentalizing. To the best of our knowledge, there is only one questionnaire widely used (available in 15 different languages) in the assessment of parental mentalization—the aforementioned PRFQ (Luyten et al., 2017). The PRFQ is designed for parents of infants up to five-year-old children to assess the parent’s capacity to treat the child as a psychological agent (Luyten et al., 2017), and it is based on the operationalization of parental reflective functioning. PRFQ consists of 18 items aiming to capture three key features of reflective functioning: (a) *interest and curiosity in mental states* (IC), (b) the ability (or inability) to recognize the opacity of mental states, i.e., *certainty about mental state*s (CMS), and (c) *pre-mentalizing modes* (PM) characteristic of parents with severe impairments in parental mentalizing.

The first, IC dimension consists of items such as *“I like to think about the reasons behind the way my child behaves and feels,”* which target the parent’s general ability to relate to the child’s mind with curious interest. The items of second, CMS dimension reflect excessive certainty and an inability to grasp the opacity of the child’s mind (e.g., *“I can completely read my child’s mind”*). A good parent’s mentalizing ability lacks such certainty. The third dimension, PM, contains items such as *“My child cries around strangers to embarrass me”* or *“I believe there is no point in trying to guess what my child feels”* that reflect gross errors in the ability to mentalize the child’s mind; either the child’s mind appears incomprehensible or the interpretations are distorted.

The PRFQ offers a way for researchers to quickly obtain an assessment of the parent’s reflective capacities in different settings (Carlone et al., 2023). Its subscales have been shown to be related to parent’s emotion regulation (Schultheis et al., 2019) and distress tolerance (Rutherford et al., 2015), and they have been successfully used in evaluating the efficacy of some interventions (Camoirano, 2017). However, the PRFQ still has its limitations. First, PRFQ does not provide an opportunity to assess self-mentalizing, i.e., the parent’s ability to reflect their *own* emotions, intentions, desires, and thoughts. This is important as self-mentalizing may be an even more important factor in quality child–parent interactions than child-mentalizing (Suchman et al., 2010). The second problem is typical to all self-assessments of mentalization; the PRFQ relies on a meta-perspective to appraise the parent’s own mental states (Stuhrmann et al., 2022). In other words, the parent must utilize their current mentalization capacity in their assessment of the very same capacity, which is questionable in terms of validity. In addition, items that are written as they are in the PRFQ, on a very general level, may especially require a meta-level evaluation. We suggest that more context-bound items (i.e., items that direct the parent to think about daily interaction situations with their child) could make the self-assessment less reliant on the meta-perspective for the parent.

The third challenge, and one that is central to the present study, relates to the sensitivity of PRFQ in capturing change in various populations. It is important to note that also parents who have capacity to mentalize their children to begin with may encounter difficulties in their mentalizing due to, for example, the child’s language impairment or neuropsychiatric problems or due to a challenging life situation. PRFQ distinguishes non-mentalizing parents and potentially a change in their ability to see their child as an intentional subject, but it may not necessarily capture subtle changes in mentalizing, especially with parents who are more able to mentalize themselves and their children. In other words, it does not give the parent the opportunity to assess how *well* they perceive themselves to be mentalizing in various everyday situations involving themselves and their child. However, this would be crucial, especially for capturing changes occurring in parent’s mentalization ability during interventions and including those parents with relatively good mentalizing skills, who can also benefit from additional support.

In our view, there is a need to develop a new, reliable, and valid instrument to measure parental mentalizing and changes therein, especially in the context of parental interventions. Parents are subjected to numerous different interventions, but to the best of our knowledge, no cost-effective measures are available to specifically assess changes in parental mentalizing. In order to broadly target the changes in parental mentalization capacity in various interventions, we believe that a new, change-sensitive questionnaire on parental mentalizing is a necessary addition to the field. The current study describes development and validation of a new questionnaire, Parental mentalizing questionnaire (PMQ). We define effective parental mentalizing as *parental mentalizing awareness* by which we refer to the parent’s ability to achieve a particular state of mind in interactions with the child, marked by: an awareness of the self and the child, including awareness of the child’s different perspective; experiences of clarity and curiosity; and flexibility in active processing of information to achieve understanding.

Our definition of parental mentalization in the PMQ follows the basic definitions of mentalization (Bateman and Fonagy, 2016) and parental reflective functioning (Slade, 2005; Luyten et al., 2017). It captures self-assessed cognitive processing during the interaction, as recalled by the parent, and accounts for the parent’s ability to reflect on their own state of mind and their efforts in imagining the child’s inner world. However, our definition is also rooted in mindfulness, which is an overlapping concept with mentalization (Choi-Kain and Gunderson, 2008). The model of mindfulness (Bishop et al., 2004) includes two components. The first component involves the self-regulation of attention so that it is maintained on immediate experience, thereby allowing for increased recognition of mental events in the present moment (Bishop et al., 2004). Heightened, sustained attention and vivid moment-to-moment observation of experience are precursors of sensitivity in understanding all mental states in oneself and in others. It is difficult, if not impossible, to reflect clearly on current or past experiences or develop understanding for the perspective of the other person without clear, sustained attention. The second component is an orientation of curiosity, experiential openness, and acceptance (Bishop et al., 2004), which is the “containing functioning” of mindfulness (Török and Kéri, 2022). Accepting attitude enables mentalization-based emotion regulation (Török and Kéri, 2022) allowing one to enter more fully and openly in one’s own and other’s subjective experience. We suggest that these two components form the basis for effective parental mentalizing; hence, they are included within our definition.

We hypothesize that parent’s capacity for mentalizing awareness can be operationalized through four separate dimensions, which are a part of a higher-order parental mentalization construct. In that line, we have created a new questionnaire for parental mentalizing, the PMQ. The present study describes the process of scale construction, including creation of items (identification of domain, operationalizing parental mentalizing, and generation of items), development and evaluation of scale psychometrics (data collection, confirmatory factor analysis), as well as a comparison with the PRFQ (Luyten et al., 2017) within this study. Our assumption is that, despite its shortcomings, PRFQ is a valid measure for distinguishing poorly mentalizing parents from those with mentalizing abilities. The new measure we have developed, PMQ, assessing parental mentalizing in a more nuanced manner, was not expected to conflict with the results of PRFQ. We expected that the PMQ, as a whole and its subscales, will correlate positively with the PRFQ’s IC factor and negatively with the PM factor. We expected negative or no correlation between the PMQ and the CMS, but we consider a weak positive correlation to be possible. This is due to the previous finding that the -CMS and PRFQ-IC factors are weakly but significantly correlated (*r* = 0.30, *p* < 0.01) (Luyten et al., 2017). According to Luyten et al. (2017), high levels of CMS and IC can be maladaptive in that they may be associated with intrusiveness and intrusive hyper-mentalizing in particular (e.g., assuming that the parent “knows” everything about their child’s mental states) (Luyten et al., 2017). We also expect to find support for our hypothesis regarding the four-factor structure with one higher-order factor via confirmatory factor analysis (CFA) of the data we collect from parents through social media and email lists.

We recruited participants who were parents of children aged two to six in Finland, and the items of PMQ have been constructed to fit interactions with a child of this age. Supporting parental mentalizing at this phase of the child’s development is particularly important. At this age, the dyadic parent–child interaction requires a new kind of imagination and perspective-taking from the parent due to the child’s cognitive and language development. Most importantly, this age is often characterized by experiences of intense emotions. It is common for toddlers to have a tantrum at least once per day (Daniels et al., 2012), and delays in development or speech/language may result in an increase in the frequency and severity of temper tantrums (Daniels et al., 2012). This can be challenging for parents, and indeed, parenting in toddlerhood can be marked by “times of emotional dysregulation in both parent and child (Havighurst et al., 2019).” At this stage, the way parents manage their own emotions and how they respond to their toddler’s emotions is important for the favorable emotional development of the child (Havighurst et al., 2019). The parent’s ability to mentalize plays an important role here, as it helps the parent to respond comprehensively to their children’s distress, enabling them to better manage negative behavior cycles during moments of child anguish (Senehi et al., 2018). Internalized regulatory processes experienced in the context of the parent–child relationship provide opportunities for toddlers to begin developing their own self-regulation skills (Senehi et al., 2018). The toddler also learns to mentalize through play, and the parent, when curious and playful enough and able to reflect on the child’s mental states in a marked manner, can help the child to begin learning how to mentalize through play (Muller and Midgley, 2020).

## Methods

### Domain identification and item development

The process of scale construction began with screening of the theoretical foundations of mentalization and various operationalizations of parental mentalization. Central assessment tools of mentalizing and parental mentalization were also reviewed. The theory of mindfulness and questionnaires measuring mindfulness were also screened. Based on these starting points, we devised an operational definition of parental mentalizing and four hypothesized factors (SELF, CHILD, E, and C) to describe parental mentalization. The first dimension, *Parental self-mentalizing* (SELF) reflects the parent’s self-assessed ability to be aware of their own mental states and to reflect on them. The second dimension, *Parental child-mentalizing* (CHILD), reflects the parent’s self-assessed awareness of the child and the child’s perspective in interaction situations. The third dimension, *Effort* (E), relates to active effort to reach and understand the child’s mind. The fourth hypothesized factor, *Curiosity* (C), is about parent’s emotional interest toward the child and child’s inner life.

We expected each of these four dimensions to load into their own factors. We also assumed that all four factors would further load on one higher-order factor (Higher) describing the parent’s mentalizing ability as a whole. Approximately twenty items were formed for each of the four dimensions, with a seven-point Likert scale (1 = “completely untrue,” 7 = “completely true”) in response options. In total, 77 items were created, following the recommendation that the item pool should be at least twice as long as the desired scale (Boateng et al., 2018). The context- and relationship-dependent nature of mentalization was taken into account in the response instructions such that parents were asked to reflect on interaction situations with one particular child (in case they have multiple) over the past 2 weeks. The instruction given to the parents was: “Before answering, take a moment to remember moments when you were with your child over the past 2 weeks. Try to recall different everyday situations: eating, playing, and other activities together. If you have several children, consider your time with only one child when answering the questions.”

### Target group and expert evaluation

Next, the scale was preliminarily evaluated by three parents from the target population via an online survey tool (Webropol). They responded to all 77 questions, after which they were asked to provide open feedback about whether the items seemed to be relevant for the parents of young children. This led to a more precise formulation of some complex items and highlighted that the formulation of the finished scale should be short and clear. After these adjustments, the items were subjected to expert judges for evaluation. Four interested experts, contacted via the Finnish Mentalization Association,^1^ were asked to evaluate all 77 items in accordance with mentalization theory. They were asked to make comments at a general level, but also to provide specific suggestions and reflections about the items. According to the feedback, the PMQ item pool assessed parents’ mentalization ability in a versatile manner. The critical feedback concerned the wording of some items, which was considered to encourage pseudo-mentalization [i.e., problematic non-mentalizing mode in adult thinking, which manifests itself as lack of a personal-emotional grounding in lived experience (Esposito et al., 2022)]. Based on this feedback, items written in the form “I could describe my thoughts, feelings, and sensations…” were changed to “I am able to describe thoughts, feelings, and sensations…” Furthermore, some unclear items were formulated more precisely, some were dropped out, and some were divided into two parts. After this process, the item pool was reduced to 69 items.

### Participants

A total of 321 participants were recruited via social media and e-mail lists. A total of 284 (88.8%) of the participants were women and 32 (10%) were men. Four (1.2%) of the respondents did not want to state their gender. In terms of educational background, the participants were divided as follows: comprehensive school 4 (1%), upper secondary vocational school or vocational course 13 (4.1%), upper secondary general school, further vocational qualification, or bachelor’s degree 31 (9.7%), and university degree 168 (52.5%). The average age of the participants was 37.4 years.

### PRFQ

Parental Reflective Functioning Questionnaire, PRFQ is a brief multidimensional self-report measure that assesses parental reflective functioning. It consists of 18 items aiming to capture three key features of parental reflective functioning that are: (a) *Interest and curiosity in mental states* (IC), (b) the ability (or inability) to recognize the opacity of mental states, i.e., *Certainty about mental State*s (CMS), and (c) *Pre-mentalizing modes* (PM) characteristic for parents with severe impairments in parental mentalizing. PRFQ factors are interpreted so that highly reflective parents would be interested in (IC) but not too certain about mental states (CMS) and show a low level of non-mentalizing stance (PM) (Stuhrmann et al., 2022). PRFQ has six items per factor and a seven-point Likert scale (1 = “strongly disagree,” 7 = “strongly agree”) as response options. Development and validation of PRFQ is reported by Luyten et al. (2017). In the present study, participants completed the PRFQ after the PMQ in the online survey. After data collection, the dimensionality of the PRFQ in relation to the collected data was confirmed using CFA. Following absolute and relative fit indices (Hu and Bentler, 1999), the model fit for the three-factor model was acceptable, with χ^2^(132) = 244.878, *p* < 0.000, RMSEA = 0.052, CFI = 0.920, TLI = 0.907, SRMR = 0.059. The estimates for internal consistency (Cronbach alpha) were 0.786 (IC), 0.858 (CM), and 0.516 (PM).

### Analytic strategy

Based on theory, we hypothesized that parental mentalization comprises four dimensions or subscales of one higher-order parental mentalizing factor: (1) Parental self-mentalizing (SELF), (2) Parental child-mentalizing (CHILD), (3) Effort (E), and (4) Curiosity (C). Therefore, our analytic strategy aimed to confirm this hypothesized factor structure. Following the model generation approach [see p.11 (Kline, 2015)], first, CFA was used separately for the items developed for each hypothesized dimension. Next, in order to select a maximum of eight best items from among the items developed to measure the corresponding dimension, item reduction was conducted in an exploratory manner within each dimension. We added all the items developed for the subscale to a CFA simultaneously, and then removed items one at a time items that (1) did not have a significant loading and (2) had the weakest standardized factor loading (<0.45) until the number of items was eight or less and an acceptable model fit was obtained. Second, after establishing a good factor structure for each dimension, the factors were combined and tested in one single CFA. Third, the higher-order parental mentalization factor was added to the model. Finally, the correlations between factors of PMQ and PRFQ and the correlations between PRFQ factors and the higher-order factor of the PMQ were examined. Estimates for internal consistencies were also calculated for all aforementioned factors using SPSS (version 27).

In all the CFA-models, Mplus statistical package was used (version 8.7), and the goodness of the fit was evaluated with the following absolute and relative fit indices (36): (1) Chi-squared test (*p* > 0.05 or χ2/df < 2: good fit); (2) the Comparative Fit Index (CFI, >0.90: acceptable; >0.95: good fit); (3) the Tucker–Lewis Index (TLI, CFI, >0.90: acceptable; >0.95: good fit); (4) root mean square error of approximation (RMSEA, < 0.06: good fit; < 0.08 acceptable); and (5) standardized root mean square residual (SRMR, < 0.05: good fit; < 0.08 acceptable).

## Results

### Item reduction

Items were examined separately within each hypothesized dimension. Thirteen items had been developed to capture Parental self-mentalizing (SELF). After removing items that did not have a significant loading (1 item) and items that had a standardized factor loading below 0.45 (5 items), SELF scale consisted of seven items. The model fit was acceptable (χ^2^(14) = 36.97, CFI = 0.93, TLI = 0.90, RMSEA = 0.07, SRMR = 0.04). Since removing the only item that still had a standardized factor loading <0.50 (item 8) led the model fit to worsen, we decided to keep it in the model. Nineteen items were supposed to measure the second dimension, Parental child-mentalizing (CHILD). We removed the non-significant items (1 item) and those with the weakest standardized factor loading (10 items) until 8 items remained. The model fit was good [χ^2^(20) = 24.84, CFI = 0.98, TLI = 0.98, RMSEA = 0.03, SRMR = 0.04].

Initially, 25 items were developed to measure the Effort (E) to reach and understand the child’s mind. However, inter-item correlations between these items and other items assessing good mentalization ability (i.e., from SELF, CHILD and C factors) revealed that the items measuring the mental effort in the E factor do not map the parent’s mentalization in the hypothesized way. The purpose of this factor was to measure the parent’s ability to actively process and act in order to understand the child. We hypothesize that this could be different from the CHILD-factor, which focuses more on observations and awareness of the child’s mind. Effort-factor contained a set of items that comprehensively mapped mental effort in interaction situations. However, as mentioned, this did not seem to fit our data. This may be due to the following reasons. First, a special effort is not necessarily required if there are no problem situations in the parent–child relationship during the 2 weeks covered by the questionnaire. In a safe attachment context and environment, the parent–child interaction can run quite easily without much effort (Fonagy and Bateman, 2019). There were also some challenges in the interpretation of the wording of some items. For example, the items of E factor were written in such a way that was either situation-specific (i.e., on a challenging situation mentalizing requires effort), such as “I have sensed that something is bothering my child and tried to figure out what it was,” or child-specific (i.e., mentalization requires effort potentially because of a challenging child), such as, “Even though I know my child, at times I have had to concentrate really hard to understand their thoughts.” It is possible that these items mapped situation-specific responses (some families have overall more challenging life) and child-specific responses (some children have more challenges, i.e., special needs). Because the purpose of the E factor as a measure of a parent’s mentalizing ability did not seem to be realized in an unambiguous way, we decided to focus on the eight items assessing *lack of effort* (also change in factor’s name: Effort, E to Lack of effort, LE) that seemed more unambiguous. After removing items that did not have a significant loading (1 item) and items that had a standardized factor loading below 0.45 (1 item), the model fit of the remaining six items was good [χ^2^(9) = 17.18, CFI = 0.97, TLI = 0.95, RMSEA = 0.05, SRMR = 0.03].

Finally, 12 items were developed to measure Parental Curiosity (C). One of these items was mistakenly worded such that it did not match the response options and was therefore dismissed. We further removed four items with the lowest standardized factor loadings, but the model fit was still not acceptable [χ^2^(14) = 70.34, CFI = 0.87, TLI = 0.81, RMSEA = 0.11, SRMR = 0.05]. Removing the next item with the lowest factor loading resulted in the model fit worsening. Therefore, we decided to examine the means and variances of the remaining items to see if some of the items are problematic. Two items had a very small variance (<0.25), as well as high skewness (2.50–6.04) and kurtosis (7.00–48.69). After removing these items, the model fit of the five-item factor was acceptable [χ^2^(5) = 12.19, CFI = 0.97, TLI = 0.94, RMSEA = 0.07, SRMR = 0.03].

Next, all four factors were evaluated in a single CFA. The model fit was not acceptable [χ^2^(293) = 519.59, CFI = 0.88, TLI = 0.86, RMSEA = 0.04, SRMR = 0.07]. The examination of the modification indices revealed that two items loaded on more than one factor. After these items were removed, the model fit was acceptable [χ^2^(246) = 376.22, CFI = 0.92, TLI = 0.91, RMSEA = 0.04, SRMR = 0.06].

In this final model, SELF factor has seven items with standardized factor loadings ranging from 0.484 to 0.672, CHILD factor has seven items with standardized factor loadings ranging from 0.483 to 0.687, LE factor has five items with standardized factor loadings ranging from 0.543 to 0.648, and C factor has five items with standardized factor loadings ranging from 0.491 to 0.832.

**Figure 1:**
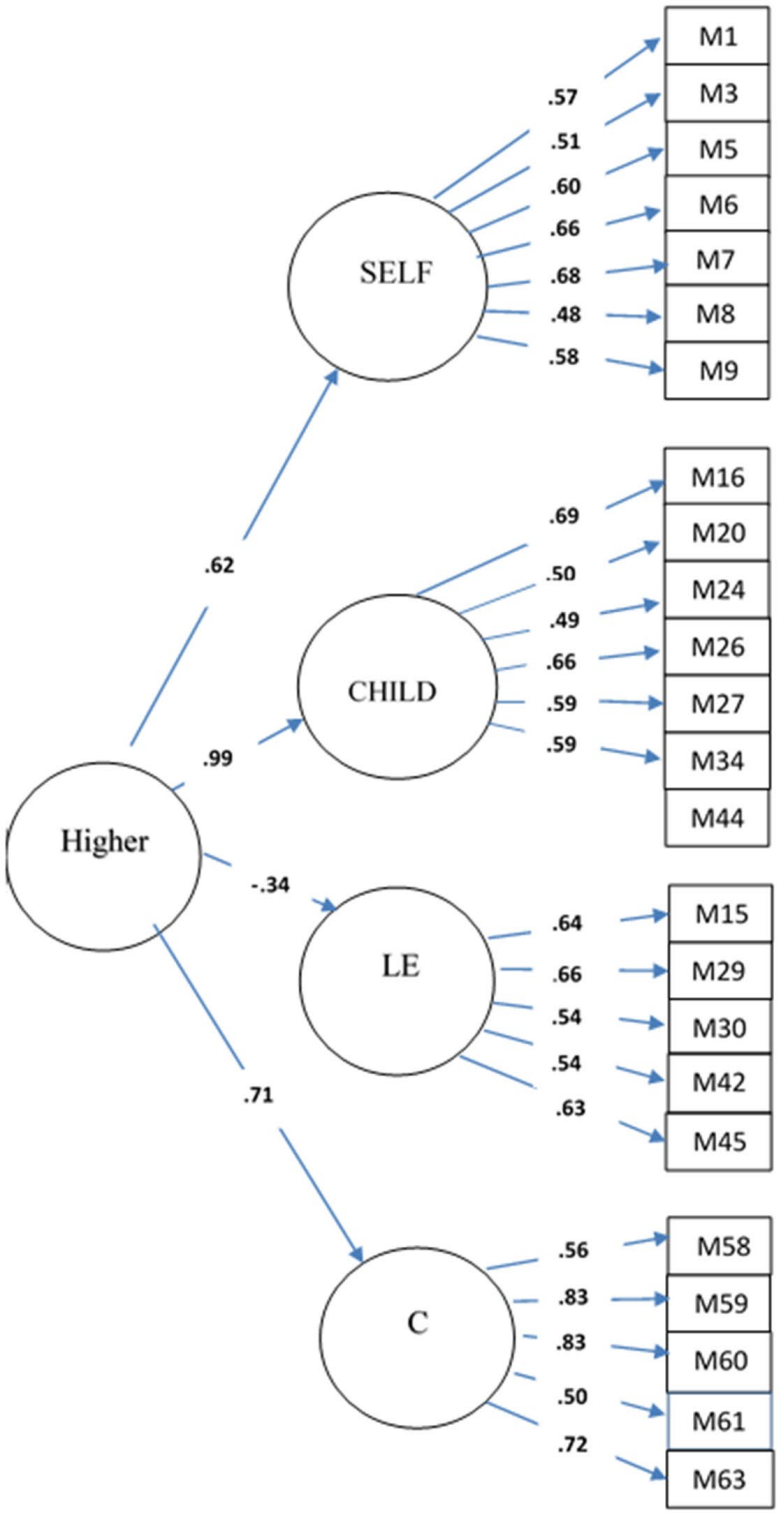
Standardized model results of CFA. Hypothesized latent constructs, Parental self-mentalizing (SELF), Parental child-mentalizing (CHILD), Lack of effort (LE), Curiosity (C), and Higher-order construct for general parental mentalizing (Higher) are presented in circles. Rectangles present measured variables (items). Bold estimates are statistically significant loadings.

Next, we added a higher-order factor to the model, and allowed all four factors to load on it. The model fit was acceptable (χ2(248) = 378.14, CFI = 0.92, TLI = 0.91, RMSEA = 0.04, SRMR = 0.06). All factors loaded significantly to the higher-order factor with standardized factor loadings 0.62 for the SELF, 0.99 for the CHILD, −0.34 for the LE, and 0.71 for C. The estimates for internal consistency (Cronbach alpha) were 0.769 (SELF), 0.764 (CHILD), and 0.724 (LE), and 0.800 (C). The PMQ (24 items) translated from Finnish to English can be found in the Supplementary material. The CFA results are displayed in Figure 1.

### The factor correlations of PMQ and PRFQ

The factor correlations of PMQ and PRFQ are reported in Table 1. As expected, the PRFQ’s IC factor positively correlated with the PMQ’s SELF, CHILD, and C factors and negatively with PMQ’s LE factor. PMQ’s Higher factor correlated strongly and positively with IC. The PRFQ’s CMS factor did not correlate, correlated negatively or correlated weakly with all PMQ factors. The PRFQ’s PM factor correlated negatively with all PMQ factors except the LE-factor. All correlations were significant apart from the four non-significant correlations between PRFQ-CMS and PMQ-CHILD, PMQ-C, PMQ-Higher, and PRFQ-IC-factors.

**Table 1:** Correlations for PRFQ and PMQ factors.

Variables	PMQ				PRFQ		
	Self	Child	LE	C	IC	CMS	PM
PMQ, SELF	–						
PMQ, CHILD	0.61^**^	–					
PMQ, LE	−0.30^**^	−0.31^**^	–				
PMQ, C	0.43^**^	0.72^**^	−0.25^**^	–			
PRFQ, IC	0.54^***^	0.81^***^	−0.41^***^	0.73^***^	–		
PRFQ, CMS	0.22^**^	−0.03	−0.24^***^	−0.05	0.08	–	
PRFQ, PM	−0.36^***^	0.29^**^	0.63^***^	−0.38^***^	−0.41^***^	−0.19^**^	
PMQ, Higher	0.62^***^	0.89^***^	−0.42^***^	0.79^***^	0.92^***^	0.07	−0.46^***^

PMQ, SELF, parental self-mentalizing; PMQ, CHILD, parental child-mentalizing; PMQ, LE, lack of effort; PMQ, C, curiosity; PRFQ, IC, interest and curiosity in mental states; PRFQ, CMS, certainty about mental states; PRFQ, PM, pre-mentalizing modes; PMQHigher, higher-order factor ^**^p < 0.01, ^***^p < 0.001.

## Discussion

In the present study, a 24-item Parental Mentalizing Questionnaire (PMQ), was developed, confirmed, and validated comparing its factors with those of another questionnaire measuring parental reflective functioning (PRFQ). The purpose of this process was to create a new research instrument sensitive enough to measure changes in parental mentalizing within interventions. Next, we will take a closer look at how well we succeeded in this task.

Our study demonstrates the PMQ, with its four-dimensional structure, is a reliable and valid measure of parental mentalization. All four hypothesized factors were confirmed by CFA after the content of one factor (Effort, E) was reconsidered. The name of the factor was also changed (“Effort, E” to “Lack of Effort, LE”). The confirmation of self- and child-mentalizing representing two distinct factors was in line with former research on reflective functioning, where self- and child-mentalizing have been found to be interdependent albeit separate dimensions (Suchman et al., 2010; Borelli et al., 2016). Consistent with our hypothesis, curiosity was established as a factor on its own.

The change in the content of the Effort factor (to Lack of Effort, LE) was a significant modification to our original conception. Contrary to what we originally planned, this factor did not measure parental mentalization involving attention, awareness, intention, effort, and imagination. Instead, it tapped into the parent’s self-evaluation of their *inability* to mentalize in interaction situations with the child. However, we argue that this subscale, with items describing acting “on autopilot” with the child or having obstacles that prevent the pursuit of the child’s mind (i.e., “My own feelings have prevented me from thinking about my child”), is a valuable part of the PMQ. In the context of interventions, the lack of effort factor offers a possibility for a parent to reflect on their obstacles of mentalizing. According to our clinical experience, these obstacles very often relate to challenging life situations, exhaustion, and stress. The reduction of these kinds of obstacles can be a key change in successful family interventions, and they are important to measure from the mentalization point of view.

In our view, the items selected for the other subscales (SELF, CHILD, and C) in the item-reduction process reflect our operationalization of parental mentalizing. Our goal was to create a questionnaire measuring the self-assessed “mentalizing awareness” in parent–child interaction. We defined it as the parent’s ability reach a certain kind of state of mind in interactions with the child which: reflects an awareness of the self and the child (including awareness of the child’s different perspective) during interactions; is characterized by the experience of clarity and curiosity; and can be flexibly changed via active processing of information to achieve understanding. Considering the general limitations of questionnaires as assessment tools of mentalizing, our findings suggest that the PMQ’s items tap into the different parts of the definition reasonably well.

In the self-mentalizing (SELF) factor, the items (see Supplementary materials) encompass the parent’s assessments of recognition of their bodily state, recognition of their mental states and ability to describe them, awareness of environmental influences on mental states, and awareness of how the child affects the parent’s mental states. The items regarding child-mentalizing (CHILD) tap into the awareness of the child’s presence, observations of the child’s mental states, reflection on the child’s mental state, recognition of the child’s different perspective, and awareness of the impact of the child’s life circumstances on the child’s mind. The curiosity factor (C) items, in turn, capture positive affect and the element of curiosity in mentalization.

The items also seem meaningful in relation to the framework of mentalization theory more broadly. They reflect several key relational strengths associated with effective mentalizing, such as impact awareness, curiosity, reflection, and perspective taking (including recognizing that others may have different mental states) (Luyten et al., 2019). We aimed to assess the parent’s capacity for effort in parent–child interactions (i.e., capacity for controlled mentalizing), which is also an important part of effective mentalizing (Luyten et al., 2019), and we achieved this goal in an unexpected manner, as mentioned in the section regarding the LE factor.

The overall comparison of the PRFQ and the PMQ offer support for the validity of the PMQ. PRFQ’s Interest and curiosity-subscale (IC) correlated clearly and significantly with the higher-order factor of the PMQ and with the subscales. PRFQ’s certainty subscale, CMS, describing overconfidence about the child’s mental states, did not correlate with the PMQ’s good mentalizing factors except with Self-mentalizing factor (SELF). This may indicate that the PMQ’s items measuring child-mentalizing and curiosity are more likely to measure genuine understanding of the child’s mind than intrusive “knowledge” of the child’s mental states. However, it should be noted that the PRFQ’s Interest and curiosity factor (IC) and Certainty factor (CMS) did not correlate in this study either, although a weak but statistically significant correlation was found in a previous study (Luyten et al., 2017). A small correlation between PMQ’s Self-mentalizing (SELF) and PRFQ’s Certainty factor (CMS) may indicate that the self-mentalizing items of PMQ tap into overconfidence in self-reflection. The subscale measuring the Pre-mentalizing modes (PM) in PRFQ correlated consistently, negatively, and significantly with the dimensions measuring effective mentalization (and with higher-order factor) and positively with the Lack of effort-factor (LE).

Are the PRFQ’s PM factor and the PMQ’s LE factor, both of which measure non-functional mentalizing, measuring the same thing; that is, do they measure the same latent construct of pre-mentalizing because they correlate so strongly with one another? In this context, it is worth noting that the estimate for internal consistency for PRFQ’s PM subscale had relatively low reliability. The internal consistencies of the PRFQ subscales have been partly low or questionable also in some previous studies (Stuhrmann et al., 2022), and one suggested reason for this is that the PM items may measure various aspects of a broad construct, and this may result in low reliability (Ye et al., 2022; Madsen et al., 2023). This might be also the case with the PRFQ’s PM factor’s correlation with the PMQ’s LE factor. Despite the positive correlation, the items of these two factors are mostly different. Four items of PM factor target two types of distorted child perception: either interpretations derived exclusively from external features or interpretations completely alienated from reality (Madsen et al., 2023). The Lack of effort factor’s (LE) items, in turn, describe acting “on autopilot” with the child or having obstacles preventing the pursuit of the child’s mind. On the other hand, PRFQ’s Pre-mentalizing modes-factor (PM) also contains two items that refer to self-assessed difficulties in entering the inner world of the child. Our guess is, then, that these two factors do not necessarily reflect the same phenomena even though they overlap. We suggest the PMQ’s Lack of effort-factor (LE) items could measure hypomentalizing but perhaps primarily symptoms caused by stress or exhaustion. However, in this respect too, these two factors may overlap as the PRFQ’s Pre-mentalizing dimension has previously been positively correlated with parenting stress (Luyten et al., 2017) To sum up: although LE correlates with PM, it does not clearly capture global impairments in mentalizing (i.e., pre-mentalizing modes).

Could it then be inferred that the PMQ is a valid measure of genuine parental mentalizing? Since the measure we used in our comparison, the PRFQ, correlates with PMQ in the manner described above, this can be inferred with certain reservations. The PRFQ has provided promising results on the convergent validity between parental reflective functioning assessed with the Parent Development Interview (RF-PDI) (Anis et al., 2020), which is a semi-structured clinical interview that taps into parents’ representations of themselves as parents, of their child, and of the relationship between them (Sleed et al., 2020). The PMQ correlates meaningfully with the PRFQ, which can be interpreted as demonstrating convergent validity. Moreover, since PMQ also provides opportunities for assessing degree differences in mentalizing and taps into self-mentalization not measured in PRFQ, one could assume it would be even more relatable to the RF-PDI. However, this would need to be investigated directly through a similar design (i.e., PMQ and its subscales’ correlations to the PDI-rated reflective functioning scores should be examined in future research). As discussed in the introduction, self-assessment measures are prone to various biases, such as social desirability bias and the problem of taking meta-perspective to appraise the parent’s own mental states. In RF-PDI, in turn, parents must describe the mental states, meaning that they are challenged to engage in actual mentalizing, and trained evaluators then score their narratives. This differs significantly from the context of the PMQ where parents assess themselves on whether they can (and how well they can) notice, identify, and describe the mental states related to recalled interactions situations. The accuracy of the assessment can therefore be dependent, except for the parents’ assessment abilities, also on the parents’ willingness to realistically assess their own mentalizing. This, in turn, is influenced by parents’ epistemic trust/mistrust regarding the research (and the intervention, if measurement is conducted in the context of an intervention), trust forming the basis for accurately assessing one’s own mentalizing.

Is the PMQ then sensitive enough to measure changes in parental mentalization in the context of interventions? Since the PMQ has been developed with this purpose in mind, this is also an important subject for future research. To address this, the PMQ should be used as a measure among other assessment tools within pre- and post-intervention tests. The sensitivity of the PMQ could then be assessed in relation to the changes observed in measures like Parental Stress Index (PSI-4) (Abidin, 2012), the Strengths and Difficulties Questionnaire (SDQ) (Goodman, 1997), Mindful Parenting Scale (IMP) (Burgdorf and Szabó, 2021), and the Mentalization Scale (MentS) (Dimitrijević et al., 2018). Changes in the PMQ scores should also be compared with pre- and post-treatment PDI-rated reflective functioning (Slade et al., 2004) scores. Parallel changes with these measures could indicate the reliability of the PMQ in measuring changes in parental mentalization. Comparison across different instruments may also refine the PMQ in relation to other operationalizations of parental mentalizing. As the PMQ was developed in consideration of mindfulness as a closely related concept (e.g., by emphasizing awareness in interactional situations), the operationalization of the PMQ differs slightly from, for example, the operationalization of parental reflective functioning.

The following limitations can be seen in our study. The answers to the items were distributed in a skewed way (i.e., the statements are often answered at the extremes; on a Likert scale of 1–7, the averages were around six), calls into consideration the discriminating ability of the scale in the current sample. However, participants of the study were mostly highly educated, Finnish, adult mothers (average age of the participants was 37.4 years). In addition, study participation was on a volunteer-basis upon seeing an invitation to participate in the study in an email or on social media. This could have skewed the distribution of responses for multiple reasons. Firstly, parental mentalization appears to favor older age. Older mothers tend to use more appropriate mind-related comments with more positive tone when interacting with their 18-month-old children when compared to younger mothers (Demers et al., 2010). Mothers may also be more interested in their children’s mental states and more confident about them than fathers (Madsen et al., 2023). However, one study conducted in a Finnish population found that differences in the fathers’ and the mothers’ “interest and wondering about the mental states and appropriateness of reasoning” may not be significant as the child gets older (Salo et al., 2021). Additionally, voluntary decision to participate in the study and complete the questionnaire, in which parents were asked to reflect on their parent–child interactions, may have subjected the study to selection bias as these respondents were likely interested in spending time thinking about their child. This all, in turn, may translate into a better ability to mentalize which may have contributed to the high average scores in the PMQ.

The homogeneity of the participants raises the question of generalizability of the results. The fact that participants are representatives of the same culture, with the majority of participants being women of a similar age (on average past young adulthood), does not justify making universal conclusions about the ultimate validity of the measure. In Finland, our sample is not particularly unusual. The average age of having the first child in Finland in 2023 was 31.2 years (Äidit tilastoissa, 2023; Isät tilastoissa, 2023). In 2020, 74 percent of the population aged 15 and over had completed a degree beyond primary education, and the number of those with higher education varies from 26 to 40% depending on the region (Tutkinnon suorittaneiden osuus väestöstä moninkertaistunut 50 vuodessa, n.d.). However, within the Finnish cultural context, could we assume that the PMQ could differentiate between those who demonstrate poor vs. effective mentalization, for instance, among young, Finnish, immigrant fathers? Clear and meaningful correlations with the PRFQ, which has been tested across different cultures and different groups of people and genders [e.g., see (Borelli et al., 2016; Anis et al., 2020)], may be indicative of similar functionality in the PMQ. On the other hand, the PMQ is a different measure from PRFQ; its items tap into different aspects of parental mentalizing compared with the items in the PRFQ. Thus, the validity of the PMQ across different contexts, where the gender, SES, and the cultural background of the respondents differ, requires further research.

Another limitation to consider is that, in the validation of the PMQ, we relied only on the PRFQ and on expert evaluation. As mentioned before, the PMQ should undergo further testing with a wider variety of measures of parental mentalization. Finally, given the partly exploratory approach in developing the PMQ with a model generation approach (Kline, 2015), a validation study (i.e., strictly confirmatory approach) to test the factor structure and the selected items in an independent sample is needed in the future. Currently, the PMQ is in the preliminary testing stage. In this phase, we consider the PMQ to be a potential new measure of parental mentalization ability. In sum, we recommend that the PMQ be utilized in future research with different population groups across various research settings, particularly in the context of interventions, so that its validity and usefulness as a self-assessed measure of parental mentalization and changes therein could be better evaluated.

## Data availability statement

The raw data supporting the conclusions of this article will be made available by the authors, without undue reservation.

## Ethics statement

Ethical approval was not required for the studies involving humans because the research is a survey. It does not collect direct personal data. The studies were conducted in accordance with the local legislation and institutional requirements. The participants provided their written informed consent to participate in this study.

## Author contributions

TTe contributed to the development of the scale and main author. TTu contributed to the statistical analysis with TTe and commenting on the text and suggestions for improvement. OL contributed to the general comments from the beginning of the scale development, critical evaluation of the text and analysis, and suggestions for improvement. CS contributed to the general comments from the beginning of the scale development, critical evaluation of the text and analysis, and suggestions for improvement.

## Footnotes

^1^ https://www.mentalisaatioyhdistys.fi/
