# Supplementary material for: A preliminary validation of PMQ—A four-factor questionnaire measuring parental mentalizing
Source: Front Psychol. 2024 Jun 12;15:1250092. doi: 10.3389/fpsyg.2024.1250092 (PMC11199881; doi:10.3389/fpsyg.2024.1250092)
Supplement: Supplementary file 1 [file Table_1.DOCX]

**PMQ items in Finnish and in English**

Factor1: Parental self-mentalizing (SELF)

1. (m1) *Koen, että osaan kuvailla omaa oloani (tunteita, ajatuksia, tuntemuksia)* / I feel like I know how to describe my state of mind (emotions, thoughts, sensations)
2. (m3) *Olen ollut hyvin perillä omasta olostani (tunteista, ajatuksista, tuntemuksista), vaikka olenkin lapseni kanssa* / Even at times when I have been with my child, I have been well aware of my state of mind (emotions, thoughts, sensations)
3. (m5) *Pystyn nyt palauttamaan mieleeni jonkin yhdessäolomme hetken ja osaan kuvailla sen hetkisiä tunteitani ja ajatuksiani* / Right now, I am able to recall a moment we spent together, and I am able to describe the feelings and thoughts I had in that moment
4. (m6) *Osaan nyt selittää, millä tavoin lapseni on yhdessä ollessamme vaikuttanut omiin tunteisiin*/ Right now, I am able to explain how my child has affected my feelings while we were together
5. (m7) *Osaan kertoa, mitä tunteita lapseni on herättänyt minussa kuluneen päivän aikana* / I am able to describe the emotions that my child has stirred in me over the course of the day
6. (m8) *Tiedän onko kehoni ollut jännittynyt vai rento* / I know whether my body has been tense or relaxed
7. (m9) *Osaan kuvata, miten arkipäivän asiat (työ, ihmissuhteet yms.) vaikuttavat olooni ollessani lapseni kanssa* / When I am with my child, I am able to describe how everyday things (such as work, relationships, etc.) affect my state of mind

Factor 2: Parental child-mentalizing (CHILD)

1. (m16) *Olen pohtinut, millä mielellä lapseni on vaikuttanut olevan ollessamme yhdessä* / I have thought about what kind of state of mind my child seemed to be in while we were together
2. (m20) *Muistan tilanteita, joissa olen havainnut, että lapseni näkee asiat eri tavoin kuin minä* / I remember situations where I noticed that my child sees things differently from the way I see them
3. (m24) *Olen yhdessä ollessamme ollut tietoinen lapseni liikkeistä ja asennoista (esim. istuuko hän mukavasti, liikehtiikö hän levottomasti)* / At times when we have been together, I have been aware of my child's movements and positions (e.g. does he /she sit comfortably, does he move restlessly)
4. (m26) *Muistan jonkin tilanteen, jossa lapseni vaikutti ymmärtävän tai oivaltavan jotakin* / I remember a situation in which my child seemed to understand or realize something
5. (m27) *Muistan nyt jonkin yhdessäolon hetken, jonka aikana huomasin lapseni tunnetilan muuttuvan* / Right now, I remember a moment when we were together during which I noticed a change in my child's emotional state
6. (m34) *Muistan yrittäneeni kuvitella, miltä lapsestani tuntuu* / I remember trying to imagine how my child was feeling
7. (m44) *Olen pohtinut, miten ympäristötekijät (esim. päiväkoti, muut lapset, stressi, fyysinen ympäristö) saattoivat vaikuttaa lapseeni ollessamme yhdessä* / I have thought about how environmental factors (e.g. daycare, other children, stress, physical environment) could have affected my child at times when we have been together

Factor 3: Lack of Effort (LE)

1. (m15) *Yhdessäollessamme olen toiminut ikään kuin automaattiohjauksella, rutiininomaisesti, ajattelematta* / At times when we have been together, I have acted as if on autopilot, routinely, without thinking
2. (m29) *Lapseni ei ole ollut mielessäni ollessamme yhdessä* / My child has not been on my mind at times when we have been together
3. (m30) *En useinkaan ole ollut tietoinen siitä, mitä lapseni on juuri sanonut tai tehnyt* / I have often not been aware of what my child has just said or done
4. (m42) *Omat olotilani ovat estäneet minua ajattelemasta lastani* / My own state of mind have prevented me from thinking about my child
5. (m45) *En ole jaksanut ponnistella ymmärtääkseni lastani* / I have not had the energy to put effort into understanding my child

Factor 4: Curiosity (C)

1. (m58) *Minusta on ollut kiehtovaa aistia lapseni läsnäolo, kun olemme tehneet yhdessä jotakin* / At times when we have been doing something together, I have found it fascinating to sense my child's presence
2. (m59) *Muistan tilanteita, joissa lapseni ajatukset ovat herättäneet minussa uteliaisuutta* / I remember situations in which my child's thoughts have piqued my curiosity
3. (m60) *Muistan tilanteen, jossa olin utelias tietämään, miltä lapsestani tuntuu* / I remember a situation in which I was curious to know how my child was feeling
4. (m61) *Kaikki lapseni ilmaisemat tunteet ovat olleet jossain määrin mielenkiintoisia* / All the emotions that my child has expressed have been interesting to some degree
5. (m63) *Lapseni ymmärtäminen jossain asiassa on ollut minulle palkitsevaa* / It has been rewarding for me to understand my child on some matter
